# The Lipid Composition of the Exo-Metabolome from *Haemonchus contortus*

**DOI:** 10.3390/metabo15030193

**Published:** 2025-03-11

**Authors:** Pablo Godoy, Behrouz Rezanezhad Dizaji, Adriana Zardini Buzatto, Laura Sanchez, Liang Li

**Affiliations:** 1 Department of Pathology and Microbiology, Faculty of Veterinary Medicine, University of Montreal, Saint-Hyacinthe, QC J2S 2M2, Canada; behrouz.rezanezhad.dizaji@umontreal.ca (B.R.D.); l.sanchezm@javeriana.edu.co (L.S.); 2 Independent Researcher and Animal Health Consultant, Montreal, QC H4A 2V2, Canada; 3 Department of Biological Sciences, University of Calgary, Calgary, AB T2N 4V8, Canada; adriana.zardinibuzat@ucalgary.ca; 4 The Metabolomics Innovation Centre (TMIC), Edmonton, AB T6G 2E9, Canada; 5 Department of Chemistry, University of Alberta, Edmonton, AB T6G 2N4, Canada

**Keywords:** *Haemonchus contortus*, lipidomics, excretory/secretory products, host–parasite interactions, signaling molecules

## Abstract

**Background/Objectives**: Metabolomic studies of different parasite-derived biomolecules, such as lipids, are needed to broaden the discovery of novel targets and overcome anthelmintic resistance. Lipids are involved in diverse functions in biological systems, including parasitic helminths, but little is known about their role in the biology of these organisms and their impact on host–parasite interactions. This study aimed to characterize the lipid profile secreted by *Haemonchus contortus*, the major parasitic nematodes of farm ruminants. **Methods**: *H. contortus* adult worms were recovered from infected sheep and cultured ex vivo. Parasite medium was collected at different time points and samples were subjected to an untargeted global lipidomic analysis. Lipids were extracted and subjected to Liquid Chromatography–Mass Spectrometry (LC-MS/MS). Annotated lipids were normalized and subjected to statistical analysis. Lipid clusters’ fold change (FC) and individual lipid features were compared at different time points. Lipids were also analyzed by structural composition and saturation bonding. **Results**: A total of 1057 *H. contortus* lipid features were annotated, including glycerophospholipids, fatty acyls, sphingolipids, glycerolipids, and sterols. Most of these compounds were unsaturated lipids. We found significant FC differences in the lipid profile in a time-dependent manner. **Conclusions**: We predict that many lipids found in our study act as signaling molecules for nematodes’ physiological functions, such as adaptation to nutrient changes, life span and mating, and as modulators on the host immune responses.

## 1. Introduction

Gastrointestinal nematodes (GINs) cause severe impacts on animal health [1]. One of the most pathogenic veterinary nematodes is the barber pool worm *Haemonchus contortus*, which mainly affects the small ruminant industry associated with grazing systems, including sheep and goats [2,3]. *H. contortus* develops part of its life cycle in the ruminant abomasum, becoming an adult worm attached to the mucosa and feeding on blood. In a heavy infection by *H. contortus*, small ruminants can be severely harmed, showing anemia, the “bottle jaw” sign due to the edema-protein loss, and a general weakening that can lead to death [3]. Moreover, *H. contortus* and other GINs have developed resistance to several anthelmintic drug classes [4,5], representing a complex problem for controlling this pathogen in ruminant farms.

Helminths such as *H. contortus* secrete several biological compounds, termed parasite-derived biomolecules (PDBMs), including proteins, short RNAs, peptides, cyclic nucleotides, extracellular vesicles (EVs) and lipid-based molecules such as hormone-like products and lipid conjugates [6,7,8,9,10,11,12]. PDBMs have been fundamentally studied for the modulation of helminth infections over the immune responses from different vertebrate hosts, particularly for proteins [13,14,15,16]. However, the excretory–secretory lipid products from helminths remain poorly explored.

The lipids secreted by parasitic helminths have been the subject of early reports [16,17,18], but their low concentrations combined with limited technologies have restricted their analytical coverage. However, the development of powerful “omics” platforms in recent years has allowed for a deeper investigation into the nature of these PDBMs [19,20]. In eukaryotic organisms, lipids are hydrophobic or amphipathic organic molecules that act as the main components of cell membranes (e.g., glycerophospholipids—PLs and sphingolipids—SLs) [21]. They also act as precursors for energy storage, further used to generate other biomolecules (for instance, in the Krebs cycle) [22] and as signaling molecules [23]. This latter function of lipids may have significant relevance for parasitic nematodes since lipids may play a role in the interaction with the host and in the crosstalk between these organisms. Several human and animal parasitic helminths have been described as releasing PDBMs comprising lipid-based molecule analogs from higher eukaryotes, such as prostaglandin F2α (PGF2α) [24,25].

Prior studies have primarily characterized the endo-metabolomic lipid profile of *H. contortus*, examining either whole organisms [26] or isolated tissues such as the digestive tract [27]. Most of the reported endo-lipidome is composed of glycerolipids and glycerophospholipids. However, the lipids secreted by veterinary nematodes are not yet thoroughly studied, and little is known about their actual function. Hence, the main objective of the present study was to identify the lipids secreted by *H. contortus*, exploring parasite-derived lipids for advancing in our understanding of their contributions to host–parasite interactions, parasite physiology, and as potential biomarkers for anthelmintic resistance.

## 2. Methods

### 2.1. Experimental Infection of Sheep with Haemonchus contortus

Approved animal ethics protocol #19-Rech-2031 authorized the use of two Dorset lambs (without a history of anthelmintics exposure) for oral infection with approximately 4000 infective larvae 3 (L3) of *Haemonchus contortus* PF strain per lamb [28]. After infection, animals were housed for 45 days. Parasite burden was monitored from day 18 post-infection, collecting fecal samples from the rectum of each animal daily and subjected to coprological examination using standard methods [29]. Once the number of *H. contortus* eggs reached an arithmetic mean of 500 eggs per gram (EPG), animals were humanely euthanized, and a necropsy was carried out to extract the abomasum of each infected animal.

### 2.2. Culture of H. contortus Adult Worms for Exo-Metabolome Collection

*H. contortus* adult worms were collected from the abomasum’s mucosa and thoroughly washed several times with PBS (Phosphate Buffer Saline). Groups of 20 individuals per replicate, including female and male *H. contortus* adult worms, were cultured in Petri dishes with RPMI medium containing Penicillin/Streptomycin and incubated at 37 °C with 5% CO_2_ [30]. Media from *H. contortus* cultures were collected and replaced at three-time points: 0 h (RPMI), 2 h, 4 h, and 8 h post-incubation. Samples were frozen and kept at −80 °C until further analysis.

### 2.3. Global Lipidomic Analysis on H. contortus Culture Medium

Six samples were sent for lipidomic analysis at The Metabolomics Innovation Centre (TMIC—University of Alberta, Edmonton, AB, Canada), including one biological replicate at 0 h—RPMI, two biological replicates collected at 2 h and 4 h, and one biological replicate collected at 8 h. The number of biological replicates was limited due to the diluted nature of culture media. These samples have low concentrations of excretory–secretory lipids, requiring many *H. contortus* worms per replicate to obtain detectable lipid signals. Given the technical constraints in collecting sufficient material, this study serves as an exploratory investigation to characterize secreted lipids and establish methodological feasibility. Future studies with increased biological replicates will validate these initial findings and improve statistical robustness.

### 2.4. Samples Preparation and Lipid Extraction

The six samples were subjected to a modified Folch liquid–liquid extraction protocol with extraction duplicates [31,32]. Samples were randomized for preparation and analysis. Technical duplicates were used to assess the repeatability and analytical precision of experimental procedures. Quality control was further performed with six technical replicates of a pooled sample. Briefly, samples were thawed in a 4 °C refrigerator and vortexed. Two aliquots of 200 μL of each sample were evaporated to dryness with a nitrogen blowdown evaporator at 24 °C. The residue was resuspended in 40 μL of water, 1.34 μL of internal standard solution (Avanti Splash Lipidomix Mass Spec Standard, Avanti Polar Lipids—a commercial mixture of 14 deuterated lipid standards, Oakville, ON, Canada) and 274 μL of methanol. The internal standard mixture volume (1.34 µL) was chosen to match the low concentration of secreted lipids in the samples, minimizing ion suppression and detector saturation while maintaining consistency with the sample matrix. Although these small volumes introduce potential pipetting errors, this was controlled using calibrated pipettes and experienced analysts to ensure reproducibility.

The mixture was extracted with 551 μL of dichloromethane, followed by a clean-up step with 166.6 μL of water [33]. Samples were then allowed to equilibrate at room temperature (RT) for 10 min and centrifuged at 16,000× *g* for 10 min at 4 °C. An aliquot of the organic layer was evaporated to dryness with a nitrogen blowdown evaporator [33]. The residue was re-suspended in 4 μL of mobile phase B (MPB—10 mM ammonium formate in 95:5 2-propanol/water), vortexed for 1 min, and diluted with 36 μL of mobile phase A (MPA—10 mM ammonium formate in 50:40:10 methanol/acetonitrile/water). The extracts were equilibrated for a minimum of 4 h at 4 °C in 250 µL polypropylene inserts placed inside 2 mL amber vials with PTFE/silicone septa caps prior to injection. All samples were injected within 36 h following extraction.

A pooled mixture of all sample was prepared for quality control (QC). Six replicates of the QC pool were extracted and injected following the same procedure described for samples to evaluate technical reproducibility. Relative standard deviations for internal standard peak intensities in these six technical replicates were between 2.5 and 23.4%.

### 2.5. Liquid Chromatography–Mass Spectrometry (LC-MS) Analysis Condition

The LC-MS analyses of lipid extracts were carried out in a Thermo Fisher Dionex UltiMate 3000 UHPLC (Thermo Fisher Scientific, Waltham, MA, USA) coupled to a Bruker Maxis II QTOF Mass Spectrometer (Bruker Corporation, Billerica, MA, USA) [34]. The 12 sample extracts (extraction duplicates of 6 samples) and 6 extraction replicates of the QC pool were injected in duplicates for positive and negative ionization (i.e., two runs per extraction in each polarity). Analysis conditions included the following: using a Waters Acquity CSH C18 column (2.1 × 100 mm, 1.7 μm), with 21.5 min gradient elution followed by 5 min of equilibration (0 min—10% MPB; 1.8 min—10% MPB; 8.5 min—30% MPB; 18 min—95% MPB; 21.5 min—95% MPB; 22 min—10% MPB), a flow rate of 250 μL/min, and ESI-QToF detection (capillary voltage of 4500 V; endplate offset of 500 V, nebulizer gas pressure of 1.0 bar, dry gas flow rate of 8.0 L/min; dry temperature of 230 °C). A 1 min segment for mass recalibration was inserted at the end of each chromatogram, during which 0.10 mM sodium formate calibrant solution was infused into the ion source using a peristaltic pump. The characteristic sodium formate clusters were further employed to correct the mass-to-charge (*m*/*z*) values detected throughout each chromatogram, improving mass accuracy. Examples of obtained chromatograms in positive and negative ionization are provided as Supplementary Information (Supplementary Figure S2).

The injection volumes selected for this study, 5 μL injection for positive ionization and 12.5 μL for negative ionization, were optimized to enhance sensitivity for low-abundance analytes while still being within the column’s capacity. Different volumes were employed for detection under positive and negative electrospray ionization due to the inherent characteristics of each polarity. Samples were prepared in initial mobile phase conditions (10% of MPB, 90% of MPA) to minimize peak broadening and precipitation effects. Chromatographic performance was monitored, and peak symmetry, retention time reproducibility, and resolution remained acceptable, indicating that the injection volumes did not compromise data quality. The separation was carried out under a column temperature of 42 °C, selected to balance viscosity reduction, pressure control, and analyte stability. Given that mobile phase B consists mostly of 2-propanol, a moderately high temperature was necessary to reduce backpressure while maintaining chromatographic efficiency.

MS and MS/MS data were acquired for ions detected with mass-to-charge ratio (*m*/*z*) between 150 and 1500 Da with MS/MS collision energies varying with precursor *m*/*z* between 20 and 40 eV (MS acquisition rate of 1.44 Hz, variable data-dependent MS/MS acquisition rate with cycle time of 1.2 s). The acquired chromatograms were processed with a Python3.9.8-based in-house developed software named NovaMT LipidScreener 1.0.0. (Nova Medical Testing, Inc., Edmonton, AB, Canada), including mass recalibration using the sodium formate segment, pick picking, alignment, adduct removal, data cleansing, and polarity merging (*m*/*z* tolerance of 20.0 ppm and 5.0 mDa; retention time tolerance of 15 s, minimum peak length of 6 consecutive spectra, filtering by detection in at least 80% of injections within at least one group). Features detected for positive and negative ionization were merged as one unique feature. When a signal was detected in both ionization modes (based on *m*/*z*, retention times, and expected adduct forms), the peak with the highest intensity was retained, and the lower-intensity peak was removed from the dataset to avoid redundancy.

### 2.6. Lipid Annotation

An automated three-tier annotation approach embedded in NovaMT LipidScreener was employed for lipid annotation [33,34]. Supplementary Table S1 shows all annotated lipids, along with their annotation levels. Tiers 1 and 2 included lipids annotated by MS/MS match to publicly available databases (MSDial LipidBlast, Human Metabolome Database—HMDB, and LC-MS/MS libraries housed at the MassBank of North America) [34]. Tier 1 annotations included MS/MS matches with a spectral similarity score of 500 and precursor *m*/*z* error ≤ 5 mDa, while Tier 2 annotations had MS/MS spectral similarity score ≤ 500 and ≥100 with precursor *m*/*z* error ≤ 5 mDa. Tiers 1 and 2 annotations are shown in Supplementary Table S2. Unfortunately, current lipid MS/MS databases do not extensively cover parasite-derived compounds. Hence, features not matched to any compounds based on MS/MS spectra were searched for mass match against a curated version of the Lipid Maps database with *m*/*z* error ≤ 5 mDa MS/MS [34,35,36].

All annotations presented in this study are putative (Supplementary Table S1). Lipids and other biological molecules can have many isomeric or isobaric forms with identical chemical formulas, masses, and similar MS/MS fragmentation patterns. MS/MS-based annotations include the definition of lipid classes and subclasses, the composition of fatty acyl/alkyl residues (or summed composition if individual residues are not specified in the source database), and functional groups. Tier 3 annotations were assigned at the species level, detailing lipid class and subclass, the total number of carbons, the total number of double bond equivalents, and the total number of additional oxygen or other atoms. The position of double bonds and the stereochemistry of compounds cannot be determined using this untargeted lipidomics platform.

While Tier 3 lipids were annotated based on accurate mass, this level of confidence alone is insufficient for definitive annotations. MS-based automated identification of lipids may result in many isomers and isobars possibly matched to each detected feature. Hence, we applied a six-tier filtering and scoring approach to restrict the number of isomers/isobars and select the best identification, as previously published [37]. All annotations were filtered for expected chromatographic retention times and adducts. For example, a triacylglycerol is expected to have high retention by the reverse-phase chromatography conditions employed for this work; hence, mass-match annotations for triacylglycerols with low retention times were excluded. Furthermore, most lipid classes are detected as adducts that depend upon modifiers added to the mobile phases and sample medium. The mobile phases and chromatographic conditions used for this work enabled adducts detection depending on the structure of each molecule, including [M + H]^+^, [M + NH_4_]^+^, [M + Na]^+^, [M-H_2_O + H]^+^, [M-H]^−^, [M + HCOO]^−^ and [M-CH_3_]^−^. However, different lipid subclasses can form different ions and adducts, e.g., simple fatty acids are often detected as [M-H]^−^ but triacylglycerols cannot be ionized by the loss of a proton, being more commonly detected in positive ion mode as the [M + NH_4_]^+^ adduct. Annotation possibilities for adducts that could not be detected were excluded (e.g., triacylglycerols as [M-2H]^2−^). When a signal was putatively matched to multiple lipids within the *m*/*z* tolerance of 5 mDa after the retention time and adduct filtering, the possibilities were ranked based on expected adducts, double-bond equivalent ratios, even or odd fatty acyl chains (i.e., odd carbon numbers are less common in nature), presence of unexpected modifications (oxidation, dehydration, heteroatoms, etc.), and expected sensitivity within the employed analytical approach (e.g., sterols have lower ionization performance, being less likely to be detected than glycerophospholipids). All isomeric and isobaric possibilities that passed initial filters for retention time and adduct detection were kept for each mass-matched feature and ranked according to the characteristics of the employed method, lipid subclass and biological fluids. Low scores were given to the most likely identification possibilities, while high scores were given for less plausible lipids. For example, *m*/*z* matches to oxidated lipids received higher scores (i.e., less likely holding the correct annotation) as most lipids in nature are not expected to contain extra oxygen atoms. The annotation with the highest probability following this scoring system was selected for statistical analysis.

### 2.7. Data Normalization

Data normalization was performed for all samples using fourteen internal standards belonging to different lipid classes (Avanti Splash Lipidomix Mass Spec Standard, Avanti Polar Lipids). The annotated lipids were matched to one of the fourteen internal standards according to lipid class similarity and the expected retention time range for each class [37]. Intensity ratios, derived from the intensity (i.e., peak height) of each lipid divided by the intensity of the matched internal standard, were calculated to normalize ion suppression, ion transmission, extraction efficiencies, and other minor changes that may occur during sample handling. The peak intensity ratios for all annotated compounds are shown in Supplementary Table S1. Peak intensities (i.e., peak heights) were selected instead of peak areas due to higher sensitivity and better reproducibility in cases where peak integration may be affected by noise, co-elution, or baseline fluctuations—common effects in untargeted lipidomics.

Background subtraction including QC lipid matches less likely to derive from nematode origin was performed to specifically pinpoint compounds derived from the parasites. The normalized peak intensities for all samples (2 h, 4 h, and 8 h) were averaged, followed by subtraction of the average normalized peak intensities for RPMI blank (0 h) and quality control replicates. Compounds with positive subtraction results were considered as parasite-derived lipid features (Supplementary Table S3).

### 2.8. Statistical Analysis

The normalized peak intensity ratios for annotated lipids (Supplementary Table S1) were uploaded to MetaboAnalyst 4.0 (https://www.metaboanalyst.ca accessed on 23 November 2023). The dataset was further auto-scaled and normalized to the median intensity ratios within each sample for statistical analysis.

Multivariate analysis was performed with Principal Component Analysis (PCA) and Partial Least Square Discriminating Analysis (PLS-DA). For univariate analysis, non-parametric volcano plots were constructed by plotting the fold change (FC) of each lipid against the FDR (false discovery rate) adjusted *p*-value (non-parametric Student’s *t*-tests with unequal variances). The FC was calculated as the ratio between normalized peak intensities for binary comparisons. The criteria for significance were an FC ≤ 0.67 or ≥ 1.5 and an adjusted *p*-value < 0.05. The Volcano plot analysis results are available in Supplementary Table S4 (2 h versus 0 h), S5 (4 h versus 2 h), and S6 (8 h versus 4 h).

## 3. Results

### 3.1. Lipids Identification

**Figure 1:**
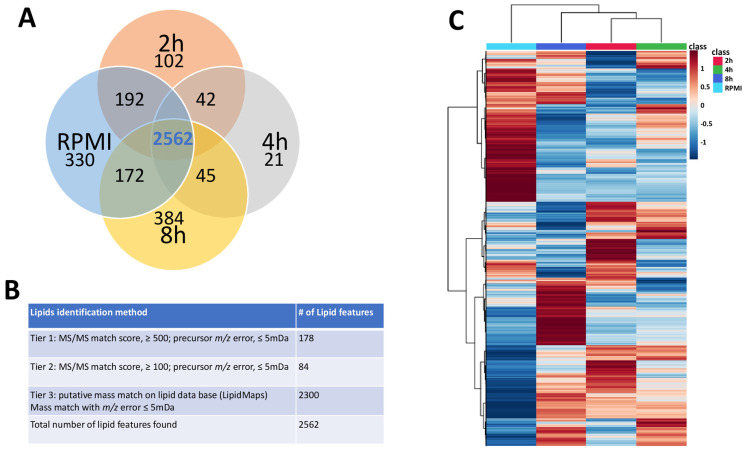
Overview of the untargeted lipidomics analysis of supernatant media from *H. contortus* adult worms cultivated ex vivo. (**A**) Venn diagram with the four time points (0 h-RPMI, 2 h, 4 h and 8 h) subjected to analysis, indicating the number of lipid features annotated on each condition. (**B**) Number of lipids annotated for each identification tier (Tier 1: MS/MS spectral similarity score ≥ 500 and precursor *m*/*z* error ≤ 5.0 mDa; Tier 2: MS/MS spectral similarity score between 500 and 100 with precursor *m*/*z* error ≤ 5.0 mDa; Tier 3: *m*/*z* match with tolerance of 5.0 mDa). (**C**) Hierarchical cluster heatmap run on MetaboAnalyst software, version 4.0. (https://metaboanalyst.ca/ (accessed on 23 November 2023)), displaying an overview of lipid features found for the 4 studied time points: RPMI/time 0 (cyan column), 2 h (red column), 4 h (green column) and 8 h (blue column). Statistical analysis was based on mean averages for normalized peak intensities per lipid feature, with a *t*-test/ANOVA and adjusted *p*-value < 0.05.

A total of 2562 compounds were annotated for the LC-MS analyses of the RPMI (time 0 h) and time point samples (2 h, 4 h and 8 h). From the three-tier ID approach, 178 lipids were annotated by MS/MS spectral match in Tier 1 (MS/MS similarity score ≥ 500 and precursor *m*/*z* error ≤ 5.0 mDa), while 84 lipids were annotated in Tier 2 (MS/MS similarity score between 500 and 100 with precursor *m*/*z* error ≤ 5.0 mDa), totalizing 262 MS/MS-annotated compounds (Supplementary Table S2). In addition, 2300 lipids were annotated with lower confidence in Tier 3 (mass-match with *m*/*z* tolerance of 5.0 mDa) (Figure 1A,B). This global set of lipid features was arranged in a hierarchical heat map for all the samples subjected to lipidomics (Figure 1C). Several clusters of lipids displayed significantly elevated or decreased intensities for each time point.

**Figure 2:**
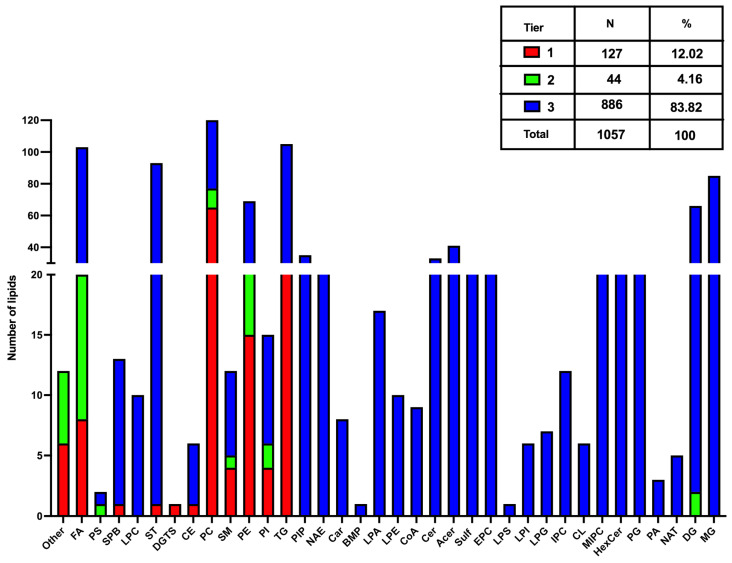
Distribution of 1057 lipids detected and annotated from *H. contortus* culture medium supernatants following subtraction of blank (media without worms, RPMI or 0 h) and quality control replicates (12 of them were categorized as “Other”, corresponding to compounds that are not classified as lipids). Compounds annotated in tier 1 have MS/MS spectral match with similarity score ≥ 500, while tier 2 have scores between 100 and 500. Tier 3 annotations are solely based on *m*/*z* match. All annotations passed a 6-tier filtering and scoring approach to handle isomeric and isobaric matches. The lipidome found in *H. contortus* culture supernatants contains several lipid sub-classes, including Fatty acids and conjugates (FAs); Phosphatidylserines (PSs); Sphingoid base-phosphates (SPBs); Lysophosphatidylcholines (LPCs); Sterols (STs); Diacylglyceryltrimethylhomoserines (DGTSs); Cholesteryl esters (CEs); Phosphatidylcholines (PCs); Sphingomyelins (SMs); Phosphatidylethanolamines (PEs); Phosphatidylinositols (PIs); Triacylglycerols (TGs); Phosphatidylinositol-phosphates (PIPs); N-acyl ethanolamines (endocannabinoids) (NAEs); Fatty acyl carnitines (Cars); Bis(monoacylglycero)phosphates (BMPs); Lysophosphatidic acids (LPAs); Lysophosphatidylethanolamines (LPEs); Acyl coenzyme A’s (CoA); Ceramides or ceramide phosphates (Cers); Acylceramides (Acers); Sulfoglycosphingolipids (sulfatides) (Sulfs); Ceramide phosphoethanolamines (EPCs); Lysophosphatidylserines (LPSs); Lysophosphatidylinositols (LPIs); Lysophosphatidylgylcerols (LPGs); Ceramide phosphoinositols (IPCs)Cardiolipins (CLs); Mannosyl-Ceramide phosphoinositols (MIPCs); Hexosylceramides (HexCers); Phosphatidylglycerols (PGs); Phosphatidic acids (PAs); N-acyl amine taurines (NATs); Diacylglycerols (DGs); Monoacylglycerols (MGs).

To evaluate the composition of the *H. contortus* exo-lipidome, we removed potential contaminants and the background from RPMI by blank average subtraction. This approach was solely performed for this data analysis step, whereas the statistical tests presented in the following sections were produced with all annotated lipids without blank subtraction. The average normalized peak intensities of blanks (RPMI without worms) and QC samples were subtracted from the average normalized peak intensities for all time point samples (2, 4 and 8 h). Compounds with higher average normalized intensities for blanks and QCs compared to time point samples were removed from the dataset (Supplementary Table S3, URL accessed on 1 December 2024: https://doi.org/10.6084/m9.figshare.24991419.v1). We found 1057 *H. contortus* excretory–secretory lipids, composed of 127 in Tier 1, 44 compounds in Tier 2, and 886 lipids in Tier 3 (Figure 2). Overall, the main lipid classes found in the lipidomics profile correspond to Phosphatidylcholines (PCs), Fatty Acyls (FAs), Triacylglycerols (TGs), Monoacylglycerols (MGs) and Sterols (STs), counting with more than 80 peaks each (Figure 2). After, there is a set of lipid subclasses ranging between 70 and 30 features each (Figure 2), including Diacylglycerols (DGs), Phosphatidylethanolamines (PEs), Acylceramides (Acers), Phosphatidylinositol-phosphates (PIPs) and Ceramides (Cers). Completing the lipidomics profile, there is broad group of lipid classes with up to 20 features on each (Figure 2), counting with Sulfatides (Sulfs), Ceramide phosphoethanolamines (EPCs), Ceramide phosphoinositols (MIPCs), Hexosylceramides (HexCers), Phosphatidylglycerols (PGs), N-acyl ethanolamines (endocannabinoids) (NAEs), Lysophosphatidic acids (LPAs), Sphingoid base-phosphates (SPBs), Ceramide phosphoinositols (IPCs), Phosphatidylinositols (PIs), Sphingomyelins (SMs), Lysophosphatidyl-ethanolamines (LPEs), Acyl CoEnzyme A’s (CoA), Fatty acylcarnitines (Cars), Lysophosphatidylgylcerols (LPGs), Lysophosphatidylinositols (LPIs), Cardiolipins (CLs), Cholesteryl esters (CEs), N-acyl amines or taurines (NATs), Phosphatidic acids (PAs), Phosphatidylserines (PSs), Diacylglyceryltrimethylhomoserines (DGTSs), Bis(monoacylglycero) phosphates (BMPs) and Lysophosphatidylserines (LPSs).

### 3.2. Lipid Classification Based on Carbon Composition

The lipid classes from all 3 tier-annotation were analyzed regarding carbon (C) structure and saturation of fatty acyl side chains (Table 1). Although the individual identification of fatty acyls from tier 3 is unlikely, most annotated lipids are unsaturated molecules (94.62% of the total lipidomics profile). We found a high proportion of the annotated lipids to contain a core structure of a very long acyl chain with more than 20 carbons in their structure (62.96%), followed by those with a long chain composed of 15–20 carbons (25.16%), and medium chain lipids with 10–14 carbons (6.56%). The saturated lipids were a minority represented by only three categories: fatty acids, glycerolipids, and glycerophospholipids. The saturated lipids with very long carbon chains (>20 C) represented just 3.7% of the global lipid profile, followed by long-chain lipids (1.73%) and medium-chain lipids (0.57%).

### 3.3. Statistical and Multivariate Analyses

The lipidomics data, including RPMI blanks and QCs (i.e., the original normalized peak intensities without the subtraction of contaminants and the background from RPMI blanks), were subjected to a hierarchical clustering analysis (dendrogram) (Supplementary Figure S1A). The QC experimental replicates were clustered, demonstrating the method reproducibility. We applied a Principal Component Analysis (PCA) 2-dimensional scores plot with quality control injections (experimental replicates of a pool of all samples) and extraction duplicates of each biological sample (0 h—RPMI, 2 h, 4 h, and 8 h) to evaluate the technical reproducibility of the dataset (Supplementary Figure S1B). The QC replicates were tightly clustered, indicating good multivariate experimental performance.

**Figure 3:**
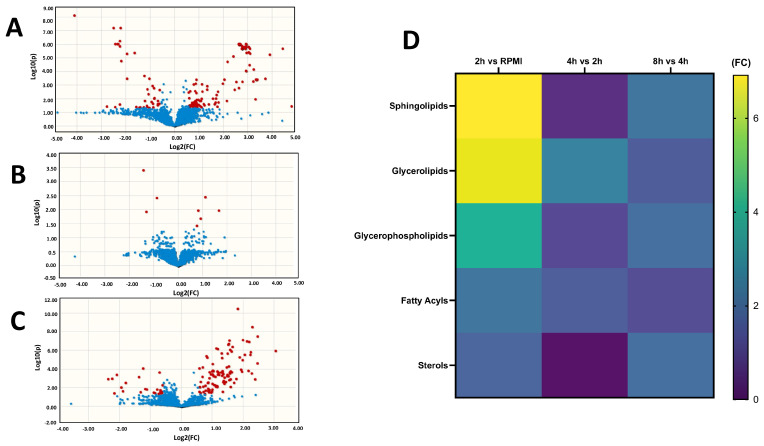
Univariate analysis based on Volcano plots (significance criteria: FC ≥ 1.5 or ≤0.67 and *p*-value adjusted for false-discovery rates-FDR < 0.05) for media collected from *Haemonchus contortus* cultures at different time points. (**A**) 2 h vs. RPMI media (time 0), showing 258 significantly altered lipids; (**B**) 4 h vs. 2 h, showing 8 significantly altered lipids; (**C**) 8 h vs. 4 h, with 126 significantly altered lipid features. In (**A**–**C**), red dots indicate number of significant lipids altered per time point. (**D**) Matrix comparing the overall means of significantly altered lipid species annotated within each lipid category.

Following data quality evaluation, we performed a univariate analysis with binary comparisons based on the fold changes (FC) and *p*-values (Mann–Whitney non-parametric tests with unequal variances corrected for false-discovery rate) of lipid features present at each time point. The normalized peak intensities for the 2562 annotated lipids (i.e., intensity detected for each lipid divided by the intensity of the most similar internal standard) were uploaded to MetaboAnalyst 4.0 (www.metaboanalyst.ca/ (accessed on 23 November 2023)). The dataset was autoscaled and normalized to the median value, followed by univariate analysis via Volcano plots for binary comparisons between (1) samples collected at 2 h versus the background RPMI (0 h) (Supplementary Table S4, URL accessed on 1 December 2024: https://doi.org/10.6084/m9.figshare.24991419.v1); (2) samples collected at 4 h versus samples collected at 2 h (Supplementary Table S5); and (3) the sample collected at 8 h versus samples collected at 4 h (Supplementary Table S6). Lipids were significantly altered for FC ≤ 1.5 or ≥ 0.67 and FDR-corrected *p*-value < 0.05. When comparing the FC between the 2 h time point samples and the background RPMI (Figure 3A), 258 lipids were significantly altered (Supplementary Table S4). For 4 h vs. 2 h (Figure 3B), we found eight lipids significantly changed (Supplementary Table S5), whereas for 8 h vs. 4 h (Figure 3C), the non-parametric univariate analysis showed 126 lipids significantly altered (Supplementary Table S6). The global FC profile for binary comparisons of lipid categories (Figure 3D) indicates a trend in the highest FC on the lipids at the 2 h time point compared with RPMI baseline (time 0), scoring the most increased FC for the Sphingolipids and Glycerolipids, follow by the Glycerophospholipids, Fatty Acyls, and Sterols. Comparing the 4 h vs. 2 h time points, there was a drop in the number of significantly altered lipids. For the 8 h vs. 4 h time points, there was an increase in the FC for the Sphingolipids, Glycerophospholipids and Sterols.

A more detailed observation of the lipids with significant FC at each time point comparison is displayed in Table 2 (mean of fold, changes, FC, ±the standard deviation, SD of altered lipids by class, where N is the number of compounds within each subclass). For the comparison between 2 h and the baseline RPMI at 0 h or FC 2 h/0 h, the lipid profile for the Sphingolipids category was composed of Sulf (N = 9, FC 7.4), followed by Cer (N = 6, FC 4.4), Acer (N = 2, FC 7.6), SM (N = 2, FC 7.5) and HexCer (N = 2, FC 7.4). In the Glycerolipids category, we found significantly altered DG (N = 12, FC 5.8) and TG (N = 7, FC 6.2). For the Glycerophospholipids category, there were PC (N = 57, FC 6.5), PE (N = 29, FC 6.5), PI (N = 7, FC 6.6), LPC (N = 3, FC 2.9), CL (N = 1, FC 3.4), PIP (N = 1, FC 3.2), and PG (N = 1, FC 1.6). For Fatty Acyls and conjugates, we found significantly altered FA (N = 26, FC 4.1), NAE (N = 14, FC 2.2), CoA (N = 2, FC 1.8), and NAT (N = 2, FC 0.3). We also found significantly altered ST (N = 18, FC 1.7).

In the FC 4 h/2 h comparison, we only found a few significantly altered lipids, including one Cer (average FC 0.5), one TG (FC 3.1), one DG (FC 1.9), LPC (N = 3, FC 1.7), and one LPE (FC 0.3). We also found one significantly altered Car (FC 1.7) and two NAE species (N = 2, FC 1.2) in the Fatty Acyls and conjugates category. For the comparison between FC 8 h/4 h, we found again a comprehensive set of significantly altered lipid features, including HexCer (N = 1, FC 3.9), EPC (N = 3, FC 2.3), SM (N = 2, FC 2.2), Sulf (N = 4, FC 2.0), Acer (N = 3, FC 1.4), Cer (N = 5, FC 0.7), DG (N = 13, FC 2.3), TG (N = 9, FC 1.7), MG (N = 2, FC 0.4), PE (N = 18, FC 3.2), PS (N = 5, FC 3.0), PC (N = 24, FC 2.6), PI (N = 2, FC 2.1), LPC (N = 2, FC 2.0), LPE (N = 1, FC 1.6), LPG (N = 1, FC 0.6), LPA (N = 1, FC 0.2), FA (N = 8, FC 2.5), NAE (N = 3, FC 0.5), Car (N = 1, FC 0.3), ST (N = 11, FC 2.0), and CE (N = 1, FC 1.8).

**Figure 4:**
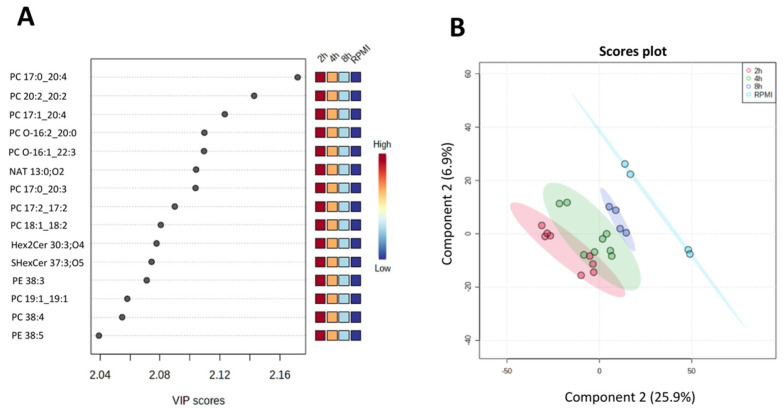
Multivariate statistics using PLS-DA for media collected from *H. contortus* culture at different time points, including 0 h (RPMI), 2 h, 4 h, and 8 h. (**A**) Fifteen most important lipids for the PLS-DA model. The side panel shows a heatmap of peak intensity ratios for these lipids, where red squares correspond to the highest intensities and blue squares, the lowest abundances. (**B**) PLS-DA scores plot (leave-one-out cross-validation R^2^ of 0.993 and Q^2^ of 0.832; permutation test with a *p*-value of 0.039 for 1000 permutations), showing the separation between the excretory-secretory lipid products at different time points, i.e., 0 h (RPMI in cyan), 2 h (in red), 4 h (green) and 8 h (blue).

We looked more closely at the lipid profile found throughout all time points with the most significant Variable Importance in the Prediction (VIP) for the PLS-DA model. Figure 4A shows the lipids with the highest VIP values, mainly PC and one NAE, HexCer, Sulf, and PE subclasses.

**Figure 5:**
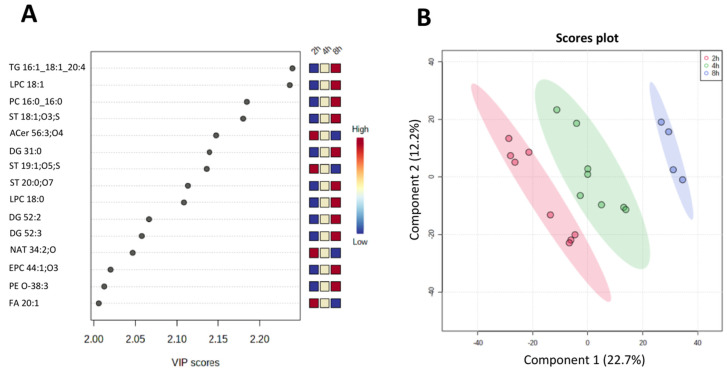
Multivariate statistics using PLS-DA for media collected from *H. contortus* culture at 2 h, 4 h, and 8 h (excluding the 0 h RPMI point). (**A**) Fifteen most important lipids for the PLS-DA model. The side panel shows a heatmap of peak intensity ratios for these lipids, where red squares correspond to the highest intensities and blue squares to the lowest abundances. (**B**). Multivariate analysis using a supervised PLS-DA model with 2 components (leave-one-out cross-validation R^2^ of 0.956 and Q^2^ of 0.782 from; permutation test with a *p*-value of 0.041 for 1000 permutations).

In contrast, when comparing only the three-time point samples without the RPMI (time 0), there is a clear separation between the time groups (Figure 5A). The most important lipids for the separation include lipids with increasing abundance from 2 h to 8 h (e.g., TG 16:1_18:1_20:4, LPC 18:1, PC 16:0_16:0, and ST 18:1;O3;S), as well as lipids with decreasing intensity ratios, indicating reduction in abundances over time (e.g., Acer 56:3;O4 and FA 20:1).

## 4. Discussion

In the present study, we have focused on characterizing the lipid profile of PDBMs released by the small ruminant parasitic nematode *H. contortus*. In our lipidomics analysis from the medium collected from *H. contortus* adult worms, we have found a diverse composition of lipids that include the main lipid classes Glycerolipids, Glycerophospholipids, Sphingolipids, Sterols and Fatty Acyls. Noteworthy is that we have discovered many fatty acyl lipids and their conjugates, such as Car, FA, NAE, NAT, and CoAs. We also found multiple novel lipids categorized as PA, PIP, BMP and Sulf. Such lipid classes have not been described before in the earlier lipidomics studies in *H. contortus* [26,27].

Throughout the diversity of the annotated lipid subclasses, we analyzed their structure based on carbon composition and saturation. We found that most of the lipids secreted by *H. contortus* correspond to unsaturated lipids (including those from Tier 3 annotation that have not definition of individual fatty acyls), with more than 65% having more than 20 carbons in their core structure, accompanied by different moieties according to each lipid class. Given that unsaturated lipids with very long chains are less soluble than saturated lipids with medium chain carbon structures, we predict that most may form micelles directly released from the worm body, conforming lipid droplets or binding to lipoprotein carriers. Also, parasitic nematodes such as *H. contortus* produce extracellular vesicles (EV) that impact their interaction with the host [15]. EVs comprise different lipids and other biomolecules surrounded by a bilayer membrane [8,38]. Work on the EVs derived from the murine GIN *Heligsomoides polygyrus* has shown the presence mainly of Glycerophospholipids, including Plasmalogens (phospholipids with a vinyl-ether bond), PS, PE, PC, LPS, LPE and LPC, and very small amounts of Sphingolipids and Sterols [39]. In another scenario, several non-soluble lipid molecules may find their way out of the parasitic nematodes through lipoprotein conjugates [40]. Studies on the characterization of the proteomic profile present in the ESPs released by *H. contortus* have described the presence of serine proteases anchored to glycophosphatidylinositols [41]. Other types of helminth-derived lipid-ligand proteins present in the ESPs are the nematode polyprotein allergens/antigens (NPA) [42]. In *C. elegans*, binding studies between NPAs and lipids have shown a high dissociation constant (K_d_) for LPA, LPE, LPC, Platelet activator factor (PAF) and Leukotrienes [42], evidencing their contribution to export lipids as part of the ESP.

Furthermore, the lipid profiling obtained in our study comprises several amphiphilic and neutral lipid subclasses characterized by one or more acyl residues attached to their core structure [43]. Among them, the most abundant lipid class in our study was triacylglycerols (TG). In higher eukaryotes such as mammals, TG is stored and released mainly from the liver as constituents of lipoproteins, including Very Low-Density Lipoproteins (VLDL) [44]. In contrast, TG transport in nematodes occurs primarily in the intestine [45]. In *C. elegans*, orthologues of mammalian lipoproteins, named vitellogenins, are involved in TG transport [46]. Vitellogenin-like proteins have also been previously described in the proteomic secretome from *H. contortus* [47]. Our findings on TG as the more abundant panel of lipids secreted by *H. contortus* are in line with previous evidence of lipid profiling composition in adult stages, as the leading group of lipids that are required as an energy source for the parasite metabolism and the main lipid constituent of eggs produced by female worms [26]. We hypothesize that most of the greater panel of TG found in our study is originated in the digestive tract [27] and released conjugated to vitellogenins from the parasite via the digestive tract, rather than TG content from female-laying eggs. The production of eggs by *H. contortus* female worms has been reported to take several days in vitro [48], excluding the possibility that the TG fraction found in our study might come from eggs present in the analyzed medium. Alternatively, TG may conform to lipid droplets (LD), along with amphipathic phospholipids, allowing the transport of TG out of the cells as described in higher eukaryotes [49]. Similarly, previous research regarding the free-living nematode *C. elegans* pointed out that LDs are produced in tissues such as the intestinal epithelium and the hypodermis [50]. Given our study’s large abundance of TG and phospholipids, we predict that *H. contortus* secretes TG from different organs and may be exported out of the parasite by two mechanisms: conjugated to vitellogenins and arranged as LD. Still, our presumptions about the function of TG once secreted by the parasite are inconclusive in their potential role in host–parasite interactions.

The remaining panel of lipids found in our study is composed of Fatty Acids, Sphingolipids and Sterols (ST). Among the latter, several articles have described that secreted ST by parasitic helminths have a role as signaling molecules modulating the host immune responses [18,51]. In addition, parasitic nematodes can produce sterol-based hormones such as ecdysteroids, most notably the 20-hydroxyecdysone, involved in the moulting through larval development to reach the adult stage [52,53]. The production and secretion of ecdysteroids in nematodes are contradictory. Experiments with radiolabeled 20-hydroxyecdysone injected in *C. elegans* have not shown the presence of this hormone in the culture medium [54]. However, similar approaches have established the secretion of this hormone by the equine parasitic nematode *Parascaris quorum* [55]. In *H. contortus*, studies have demonstrated the presence of 20-hydroxyecdysone at different life stages, mainly present in the 4th larval stage and female adult worms, implying its role in moulting and gonadogenesis [56]. Although this latter study did not address the presence of this ecdysteroid in the culture media, we cannot rule out its excretion by *H. contortus* as documented in other parasitic nematodes.

Further, extensive research has exposed that nematodes cannot synthesize de novo Sterols due to the lack of genes involved in the enzymatic pathway, as in mammals [57]. Hence, these lipids have a dietary origin or are produced by the intestinal microbiota [57]. However, detailed studies in *C. elegans* regarding the structure of excretory cells have found the antibody binding to dehydroergosterol (DHE), suggesting the production of this sterol hormone as a signaling molecule, possibly for mating purposes [58]. Our results are consistent with the hypothesis that parasitic nematodes such as *H. contortus* secrete sterol lipid hormones, which may constitute signaling molecules involved in reproduction and developmental growth.

Our work’s third most abundant panel of lipids corresponds to Sphingolipids. Among this lipid category, there are Ceramides and their conjugates (Cer), SM and Sulf. In eukaryotes, Sphingolipids have been described as components of lipid rafts, in cellular membranes that allow protein trafficking from endoplasmic reticulum to organelles and plasma membrane, and as signaling molecules for different cellular metabolic processes [59]. A well-characterized Sphingolipid in eukaryotes is the Sphingosine-1-phosphate (SP1), with pleiotropic functions involved in homeostasis and oxidative stress response [60]. In *C. elegans*, SP1 has been localized in tissues including the intestine, hypodermis and the excretory canal [61]. Moreover, long-chain Cer have been described to play a role in autophagy as a response to life-span extension in *C. elegans* [62]. Given that in our study, we kept the *H. contortus* adult worms under standard in vitro culture conditions, without the usual dietary sources provided by the ovine host, it is reasonable to presume that many long-chain Sphingolipids found in our study may correspond to signaling molecules released by the parasite to communicate between nematodes and trigger a potential function to extend lifespan over ex vivo conditions.

Intriguingly, the panel of Sphingolipids found in our study highlights a reasonable number of Sulf (N = 22, Table 1), a lipid class not yet described in the *H. contortus* lipidomics studies [26,27]. This kind of glycosphingolipids containing a sulfur radical has been barely studied in parasitic nematodes and their model organisms. Earlier studies have shown that the binding of Sulf to a particular group of glycoproteins termed “saposins” is determined by the pH of the medium, with some saposins showing a higher binding for Sulf at acidic pH [63]. Saposins have been characterized in the lipid profile from the ruminant’s parasitic nematode *Trichuris globulosa* [64]. These authors described that Sulf and Gangliosides (neural Sphingolipids) might form complexes in the parasite surface and recognize the host immune responses, acting as effector molecules to fit the parasite’s neural and metabolic networks [64]. Since *H. contortus* and *T. globulosa* have different niches in the host’s digestive tract, our findings regarding the Sphingolipids sub profile suggest differences between these two nematode species and how these lipid effectors may impact the host–parasite interactions.

The lipidomics profile found in our study is completed with fatty acids and conjugates. In this category, the most abundant lipids correspond to FA and NAE. The FA subset is mainly composed of polyunsaturated long-chain fatty acids (PUFAs), described in several helminth species, as signaling molecules to attract adult worms during mating [19] and as precursors of immune-modulatory lipids at the host–parasite interplay [7,65]. Among the saturated FA, the findings of significant folds of Stearic acid (FA 18:0) and Palmitic acid (FA 16:0) present in the lipidome profile released by *H. contortus* (See Supplementary Table S1) are consistent with previous work on the lipid features found in the metabolites secreted by parasitic nematodes [8,16], describing these two saturated FA as anti-inflammatory effectors on the host immune responses [66]. Furthermore, the presence of NAE in the lipidomics profile secreted by *H. contortus* is also a novelty compared with most previous studies on the exo-metabolomes from parasitic nematodes [7,14]. NAE has been studied in *C. elegans*, linking their function as signaling molecules to extend life span over dietary restriction [67]. In parasitic nematodes, only one work on the murine helminth *Nippostrongylus brasilensis* has shown NAE-derived lipids, including Anandamide (AEA) and docosa-hexaenoyl-ethanonalime (DHEA), suggesting an anti-inflammatory effect to modulate the host immunity during the helminth infection [68]. Collectively, our findings on *H. contortus*-derived NAE may indicate their role as signalling molecules between nematodes, involved in extending the longevity of the parasite over in vitro conditions, circumstances of dietary changes compared with those supplied by the host, and as part of the repertoire of lipid modulators on the host Th-2 response to haemochosis [11].

The profile of Fatty acyls in the present study is completed with a Car, CoA, and NAT. In invertebrates, including *C. elegans*, NAT and NAE are endocannabinoid signaling molecules involved in feeding behavior, motor control and cholesterol mobilization [69]. Recent work has shown that carnitine-esterified-PUFAs have been up-regulated in *C. elegans* when worms have been under starvation conditions [70]. This life extension process may correspond to a paracrine effect of NAEs over nematode GPCRs [71]. Altogether with these antecedents, *H. contortus* worms may secrete different Fatty acyl-signaling lipids in response to physiological adaptations when worms are cultured in vitro.

We further assessed the lipids fold change (FC) across different time points, finding that the lipidomics profile secreted by *H. contortus* varies over time. Overall, Sphingolipids, Glycerophospholipids and Sterols showed the most significant FC at 2 h, decreasing at 4 h and then again, increasing their FC at 8 h. In contrast, Fatty Acyls and Glycerolipids have a downward gradient FC from 2 h, 4 h and 8 h (Figure 4B. This trend in variation of the lipid FC released by *H. contortus* across the different time points relies perhaps on the function of these lipids in parasite physiology and adaptation to a different environment ex vivo. Still, many lipids such as PC, PE and ST found with a significant FC have also been described as effectors in the host–parasite interactions [7,14,68].

Noteworthy is that among the sphingolipids, the Sulf showed the most abundant panel upregulated at 2 h (FC 7.4, N = 9, Table 2. This finding is exciting for a few reasons: this work represents the first report of Sulf lipids secreted by a parasitic nematode, such as *H. contortus*. Only in the predatory free-living nematode, *Pristionchus pacificus* the secretion of sulfolipids has been described as signaling cues to detect its niche competitor *C. elegans*, inducing on this latter a defensive response to avoid predation from *P. pacificus* [72]. Although the Sulf found in our study were in the Tier 3 dataset from our lipidomics analysis, corresponding to mass-match identifications, our work on lipids derived from *H. contortus* provides a different profile of sulfolipids conjugated with a monosaccharide in their structure (Sulf), not previously described in the sulfolipids secreted by *P. pacificus* [72]. This remarkable discovery of Sulf derived from *H. contortus* will need further characterization to identify their detailed structure and understand their function as signaling molecules in nematode communications and at the host–parasite interactions.

The Fatty Acyl category panel highlights the presence of a mid-chain unsaturated lipid annotated as FA 14:1;O4 with FC (2 h/0 h) of 28.0 (See Supplementary Table S4). It corresponds to an isoform of the tetradecanedioic acid (TA), a lipid metabolite described in eukaryotes such as the fruit fly *Drosophila melanogaster*, where tetradecanedioate (analog of TA) was found up-regulated in the body of fasted flies compared with those refed with a sugar diet [73] among the lipid biomarkers altered by feeding restriction. This antecedent is congruent with our study and may explain the up regulation of TA in the panel of lipids secreted by *H. contortus* in vitro culture, confirming that most of the FA found in our study may be linked as signaling molecules released by the parasite when is challenged to dietary limitations. In the small panel of lipids upregulated at 4 h, we found a varied set of lipids categorized as Glycerophospholipids (e.g., LPC, LPE) and FA conjugates including Car (Table 2). LPC has been described as an immune modulator of the Th2 response in the context of allergy and inflammation [74], whereas LPE may play a signaling molecule coupling to GPCRs [75]. In the lipid panel upregulated at 8 h, we also saw a distinctive composition of lipid classes, including Cer, PE, PS, PC, FA and ST. The variation in the lipid profile measured at different time points found in our study may be explained by the presence of both female and male worms together in our experimental setting. Subsequent studies should address the study of the gender-specific lipidomics profile secreted by *H. contortus* female or male worms confirming or showing differences in the composition of the lipid profile derived from this important veterinary nematode.

## 5. Conclusions

Our study has investigated through an untargeted lipidomics analysis the profile of lipids secreted by *H. contortus* as part of the parasite-derived biomolecule repertoire. We found a large set of lipid classes released in a time-dependent manner by this helminth, which may have functions in parasite physiological responses and in its interaction with the host. Further research should investigate the lipids that are essential for the parasite to establish itself in the host and modulate the immune responses. This research provides novel information on lipid molecules that are important for *H. contortus* and possibly other parasitic nematodes that should be explored as novel targets for drug development in the control of these pathogens. Also, we have found interesting candidate lipids, such as sulfatides, that can be exploited as biomarkers for anthelmintic resistance. Comparing the profile of sulfatides produced by drug-resistant and susceptible strains may provide molecular markers for anthelmintic resistance in *H. contortus* and other parasitic nematodes that affect both human and animal health.

## 6. Limitations of the Study

Although most of the lipids found in this study correspond to annotations based on mass matches (Tier 3 dataset), the lipid profiles described here are similar to earlier lipidomics studies in nematodes focused on the somatic and secreted lipid profiles [18,24,25,26,27]. Still, a reference lipid database and specific lipid standards for free-living and parasitic nematodes are not yet available to support the lipid identities derived from these organisms. Further lipidomics research in parasitic helminths should include an integrative “multi-omics” approach to decipher the mechanisms involved in lipid production and the functional role of these bioactive molecules for these helminth pathogens [20].

An important constraint for excretory–secretory product studies is the amount of lipids isolated from parasitic nematodes. The characterization of these molecules requires substantial parasite material, which is not always available due to the necessary use of animals as hosts to obtain adult stages of GINs. For this reason, this study has a limited number of biological replicates. Also, the selection of the medium and culture conditions is crucial for lipidomics studies in adult worms; thus, careful analysis of lipid profiling should consider any bias from nutrition and parasite viability [21,76] to properly validate the dataset acquired from the lipidomics analysis.

## Figures and Tables

**Table 1:** Fatty acyl distributions for excretory–secretory lipid products found in the medium from *H. contortus* adult worms.

	Saturated			Unsaturated		
	Medium Chain (10–14 C)	Long Chain (15–20 C)	Very Long Chain (>20 C)	Medium Chain (10–14 C)	Long Chain (15–20 C)	Very Long Chain (>20 C)
Fatty Acyls						
FA	2	10	14	11	35	31
NAE	0	0	1	2	6	18
Car	0	0	0	3	2	3
NAT	0	0	0	3	0	2
CoA	0	0	6	0	0	3
Glycerolipids						
DG	0	0	1	0	4	61
TG	0	0	1	0	23	81
MG	0	2	1	33	24	25
DGTS	0	1	0	0	0	0
Sterols						
ST	0	0	0	0	43	50
CE	0	0	0	0	4	2
Glycerophospholipids						
CL	0	0	0	0	0	6
PA	0	0	0	0	0	3
PC	0	0	3	4	69	44
PE	0	0	1	0	22	46
PS	0	0	0	0	0	2
PI	0	0	0	0	5	10
PIP	0	0	2	0	0	33
PG	0	0	2	0	0	26
LPA	3	0	0	6	4	4
LPC	1	5	0	1	2	1
LPI	0	0	0	2	0	4
LPE	0	0	0	0	3	7
BMP	0	0	0	0	1	0
LPG	0	0	0	1	1	5
LPS	0	0	0	0	0	1
Sphingolipids						
SPB	0	0	0	1	12	0
Cer	0	0	0	0	0	33
Acer	0	0	0	0	0	41
Sulf	0	0	0	0	0	22
HexCer	0	0	0	0	0	28
SM	0	0	0	1	3	8
EPC	0	0	0	0	0	24
IPC	0	0	0	0	0	12
MIPC	0	0	0	0	0	22
Total	6	18	32	68	263	658
Grand Total = 1045 (100%)	0.6%	1.7%	3.1%	6.5%	25.2%	63.0%

**Table 2:** Fold change comparison of lipids secreted by *H. contortus* at different time points.

Lipid Subclass	2 h vs. RPMI (0 h)		4 h vs. 2 h		8 h vs. 4 h	
	Fold Change (Mean ± SD)	N	Fold Change (Mean ± SD)	N	Fold Change (Mean ± SD)	N
**Sphingolipids**						
Sphingomyelins (SMs)	7.5 ± 0.2	2			2.2 ± 0.9	2
Ceramides (Cers)	4.4 ± 3.7	6	0.55	1	0.7 ± 0.4	5
Acylceramides (Acers)	7.6 ± 0.0	2			1.4 ± 0.7	3
Hexosylceramides (HexCers)	7.4 ± 0.0	2			3.9	1
Ceramide phosphoethanolamine (EPC)					2.3 ± 1.8	3
Sulfatides (Sulfs)	7.4 ± 0.0	9			2.0 ± 0.6	4
**Glycerolipids**						
Triacylglycerols (TGs)	6.2 ± 0.6	7	3.1	1	1.7 ± 0.5	9
Diacylglycerols (DGs)	5.8 ± 3.4	12	1.9	1	2.3 ± 0.7	13
Monoacylglycerols (MGs)					0.4 ± 0.0	2
**Glycerophospholipids**						
Phosphatidic acid (PA)	1.5	1				
Phosphatidylcholines (PCs)	6.5 ± 0.2	57			2.6 ± 1.3	24
Phosphatidylinositols (PIs)	6.6 ± 1.8	7			2.1 ± 0.9	2
Phosphatidyl-inositol phosphates (PIPs)	3.2	1				
Phosphatidylethanolamines (PEs)	6.5 ± 1.5	29			3.2 ± 1.0	18
Phosphatidylserines (PSs)					3.0 ± 1.1	5
Phosphatidylglycerolipid (PG)	1.6	1				
Lysophosphatidylcholines (LPC)	2.9 ± 4.6	3	1.7	1	2.0 ± 0.0	2
Lysophosphatidic acid (LPA)					0.2	1
Lysophosphatidylethanolamine (LPE)			0.3	1	1.6	1
Lysophosphatidylglycerol (LPG)					0.6	1
Cardiolipin (CL)	3.4	1				
**Fatty Acyls**						
Fatty Acids and conjugates (FAs)	4.1 ± 5.5	26			2.5 ± 1.6	8
Fatty Acyl carnitines (Cars)			1.7	1	0.3	1
N-acyl taurines (NATs)	0.3 ± 0.0	2				
N-acyl ethanolamines (NAEs)	2.2 ± 4.1	14	1.2 ± 1.2	2	0.5 ± 0.2	3
Fatty acyl CoEnzyme A (CoA)	1.8 ± 0.0	2				
**Sterol Lipids**						
Sterols (STs)					1.8	11
Cholesteryl esters (CEs)	1.7 ± 1.0	18			2.0 ± 2.0	1

Footnote: FC (mean of fold changes for all lipids within each subclass), SD (standard deviation if more than one lipid was annotated within the same subclass), and N (number of lipids per subclass).

## Data Availability

All datasets included in this article are publicly available here: URL accessed on 1 December 2024: https://doi.org/10.6084/m9.figshare.24991419.v1.

## Supplementary Materials

The following supporting information can be downloaded at: https://www.mdpi.com/article/10.3390/metabo15030193/s1. Supplementary Materials are the following files: Supplementary Table S1: annotated and normalized lipid feature. Supplementary Table S2: Excretory–secretory lipid products of *Haemonchus contortus* annotated through MS/MS spectral match. Supplementary Table S3: Excretory–secretory lipid products of H. contortus after subtraction of the background (RPMI—time 0 and quality control (QC) samples). Supplementary Table S4: Significant altered lipids for 2 h vs. RPMI. Supplementary Table S5: Significant altered lipids for 4 h vs. 2 h. Supplementary Table S6: Significant altered lipids for 8 h vs. 4 h. Supplementary Figure S1A,B: Statistical analysis on medium collected samples from *Haemonchus contortus* adult worms at different time points, subjected to global untargeted lipidomics analysis. Supplementary Figure S2: Examples of chromatograms obtained in (**A**) positive and (**B**) negative ionization modes for the quality control pooled sample.
