# Supplementary material for: The Lipid Composition of the Exo-Metabolome from Haemonchus contortus
Source: Metabolites. 2025 Mar 11;15(3):193. doi: 10.3390/metabo15030193 (PMC11944095; doi:10.3390/metabo15030193)
Supplement: Supplementary file 1 [file metabolites-15-00193-s001.zip › grams obtained in (A) positive and (B) negative ionization modes for the quality control pooled sample.pdf]

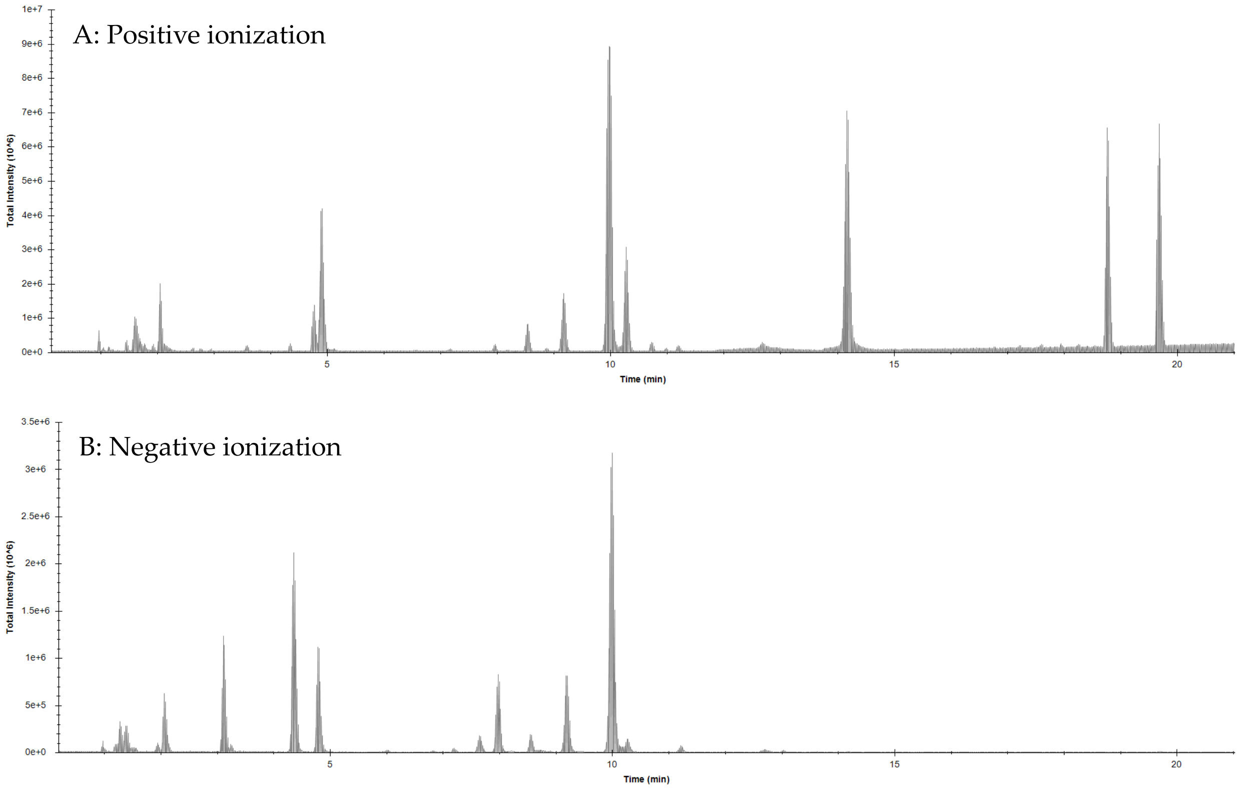

Supp. Figure S2. Examples of chromatograms obtained in (A) positive and (B) negative ionization modes for the quality control pooled sample.
