# Supplementary material for: The Lipid Composition of the Exo-Metabolome from Haemonchus contortus
Source: Metabolites. 2025 Mar 11;15(3):193. doi: 10.3390/metabo15030193 (PMC11944095; doi:10.3390/metabo15030193)
Supplement: Supplementary file 1 [file metabolites-15-00193-s001.zip › Supplementary Figures S1A and S1B.pdf]

Additional file 7. Supplementary Figures S1A and S1B.

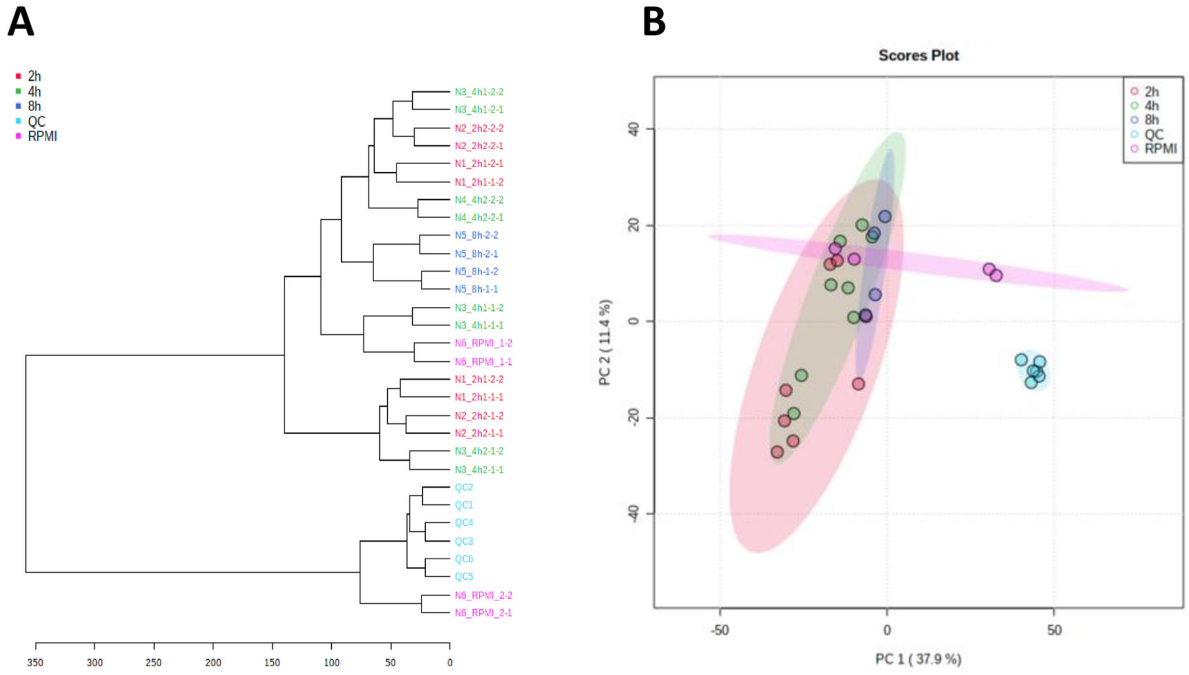

Supp. Figure S1. Statistical analysis on medium collected samples *Haemonchus contortus* adult worms at different time points, subjected to global un-target lipidomics analysis. **A**. Dendogram analysis with hierarchical clustering, including replicates from time points samples at 2h, 4h and 8h, RPMI medium (time point 0), and quality control (QC) samples, showcasing the reproducibility of the method. **B**. Principal component analysis (PCA)-2 dimensional scores, including time point samples (RPMI background medium representing time 0h in light rose, 2h clusters at 2h in dark rose, 4h clusters in blue, 8h clusters dark blue) and QC clusters far distant in light blue.
